# A novel CBCT-based method for derivation of CTV-PTV margins for prostate and pelvic lymph nodes treated with stereotactic ablative radiotherapy

**DOI:** 10.1186/s13014-017-0859-z

**Published:** 2017-08-04

**Authors:** Ciara A. Lyons, Raymond B. King, Sarah O.S. Osman, Stephen J. McMahon, Joe M. O’Sullivan, Alan R. Hounsell, Suneil Jain, Conor K. McGarry

**Affiliations:** 1 Centre for Cancer Research and Cell Biology, Queen’s University Belfast, Belfast, BT7 1NN UK; 2 Clinical Oncology, Northern Ireland Cancer Centre, Belfast City Hospital, Belfast, UK; 3 Radiotherapy Physics, Northern Ireland Cancer Centre, Belfast City Hospital, Belfast, UK

**Keywords:** SABR, Stereotactic radiotherapy, Margin derivation, Prostate cancer, Elective nodal irradiation, Multiple isocentric targets

## Abstract

**Background:**

Traditional CTV-PTV margin recipes are not generally applicable in the situation of stereotactic ablative radiotherapy (SABR) treatments of multiple target volumes with a single isocentre. In this work, we present a novel geometric method of margin derivation based on CBCT-derived anatomical data.

**Methods:**

Twenty patients with high-risk localized prostate cancer were selected for retrospective review. Individual volumes of interest (prostate, prostate and seminal vesicles and pelvic lymph nodes) were delineated on five representative CBCTs and registered to the planning CT using two registration protocols: bone match or prostate-based soft tissue match. Margins were incrementally expanded around composite CTV structures until 95% overlap was achieved.

**Results:**

CTV-PTV margins of 5.2, 6.5 and 7.6 mm were required for prostate, prostate and seminal vesicles and pelvic lymph nodes respectively using a prostate matching protocol. For the prostate and seminal vesicle structures, margins calculated using our method displayed good agreement with a conventional margin recipe (within ±1.0 mm).

**Conclusions:**

We have presented an alternative method of CTV-PTV margin derivation that is applicable to SABR treatments with more than one isocentric target. These results have informed an institutional trial of prostate and pelvic nodal SABR in men with high-risk localized prostate cancer.

**Electronic supplementary material:**

The online version of this article (doi:10.1186/s13014-017-0859-z) contains supplementary material, which is available to authorized users.

## Introduction

Stereotactic ablative radiotherapy (SABR) is increasingly used for the treatment of prostate cancer (PC), which is sensitive to larger fraction size due to a low α/β ratio [1–4]. Improved accuracy in treatment delivery, particularly since the widespread adoption of cone-beam CT (CBCT), has enabled reductions in CTV-PTV margins, facilitating dose escalation while also reducing the risk of toxicity [5–7]. Geometric accuracy is particularly important in the setting of SABR. Due to the high fractionation dose, steep dose gradients and smaller margins, a geographic miss in a single fraction could lead to considerable target under-dosing and an increased risk of toxicity [1, 8, 9].

To date, the majority of prostate SABR evidence has been for low- to medium-risk groups, where generally the prostate alone is treated [2–4]. There is a relative paucity of data regarding the use of SABR in men with high-risk prostate cancer, who potentially have the most to gain from dose escalation [10]. For this patient cohort elective pelvic nodal irradiation (ENI) is delivered in many centres, for patients treated with conventionally fractionated radiotherapy [11]. Results from a phase I/II 5-fraction SABR trial, where 25Gy was delivered to pelvic node CTVs simultaneously with a 40Gy prostate CTV prescription dose, indicated that the treatment was well tolerated in the acute setting with further follow-up data expected in the future [12].

Our centre is also currently recruiting to a high-risk prostate SABR trial with an ENI arm, where 50% of patients are prescribed 25 Gy to a pelvic node PTV, delivered simultaneously with 36.25Gy to the prostate PTV over 5 fractions [13]. However, the use of conventional margin recipes in the situation of SABR for elective pelvic nodal irradiation is potentially suboptimal, as they rely on a number of assumptions that are not met in this scenario. A fundamental assumption that is unrealistic in this case is that the target’s geometry is typically modelled as a rigid sphere. Not only is the shape of the pelvic nodal CTV structure overtly complex, but it is also subject to considerable daily variation that is dependent on bladder filling as well as other parameters [14–17].

Conventional CTV-PTV margin calculations are also not easily applicable to irradiation of multiple targets via a single treatment isocentre. For example, the prostate and lymph node CTV structures are known to move independently relative to each other, with displacements of up to 6 mm reported [14]. Additional consideration must therefore be given when employing an image guidance regime that only matches to the primary target site (e.g. the prostate).

This paper describes a composite volume approach that allows derivation of margins for two or more separate CTVs treated using a single isocentre; in this case, the prostate (PO), prostate and seminal vesicles (PSV) and pelvic lymph nodes (LN). This composite volume method was used to calculate individual margins for each structure, with the PO and PSV margins subsequently compared to PO/PSV margins derived using a commonly employed conventional statistical method [18].

## Methods

### Patients, treatment planning and delivery

Twenty patients, previously treated with conventionally fractionated radical radiotherapy to the prostate and pelvis, were selected for this retrospective review. Each patient had planning CT (pCT) images acquired using a helical CT-simulator (512 × 512 field of view, 1 mm axial pixel resolution, 2.5 mm slice width). All patients were instructed to empty their bladder and to drink 500 mL of water and had a micro-enema (Micralax®) administered prior to their pCT and each treatment session. The pCT images were imported into Eclipse™ v13.5.35 (Varian Medical Systems, Palo Alto, CA) for contouring target and organ at risk (OAR) volumes. Target volumes of interest (PO, PSV and LN) were individually delineated following a previously described pelvis IMRT protocol [19].

All treatments were delivered using a Varian TrueBeam™ linac (Varian Medical Systems) with kV-based CBCT on-board imaging. Patient set-up and CBCT verification imaging were carried out as per our institutional protocol: images were acquired following set-up for the first three fractions of treatment and on a weekly basis subsequent to this. For treatment delivery, online registration of CBCT images to the pCT was performed using the patient’s bony anatomy as a surrogate for the PTV; however, these registrations were not used in this investigation.

### Image registration

Additional offline registrations of the CBCT images to the pCT were performed independently to the online registration by a single clinical oncologist (CL), using the registration workspace within Eclipse™. Only translational shifts were considered in all cases. Two image matching protocols were studied – bone (bony pelvis) and soft tissue (ST, prostate-based), resulting in two separate datasets for analysis. Every match pair was performed in the same order: in all cases, an automated pelvic bone match was carried out first, this was followed by a separate automated ST match using the prostate as the common reference, with the resulting registration manually adjusted where required.

### Contouring

Five CBCT image sets were selected for each patient (to reflect commonly used SABR fractionation schedules) [12, 20]. To generate an appropriate representation of the variation in individual patient anatomy throughout their entire treatment, the first and last CBCT image sets were selected for each patient, with the remaining 3 CBCT image sets evenly sampled across the patient’s treatment schedule. CBCT image sets with optimum image quality were selected for analysis to ensure accuracy in the soft-tissue structures delineated and image sets of insufficient image quality were excluded from the study. Following registration of the CBCT images to the pCT, structures of interest were contoured manually in Eclipse™ by a single clinical oncologist (CL) and peer-reviewed by a second clinical oncologist (SJ).

### Composite volume generation and overlap analysis

**Fig. 1:**
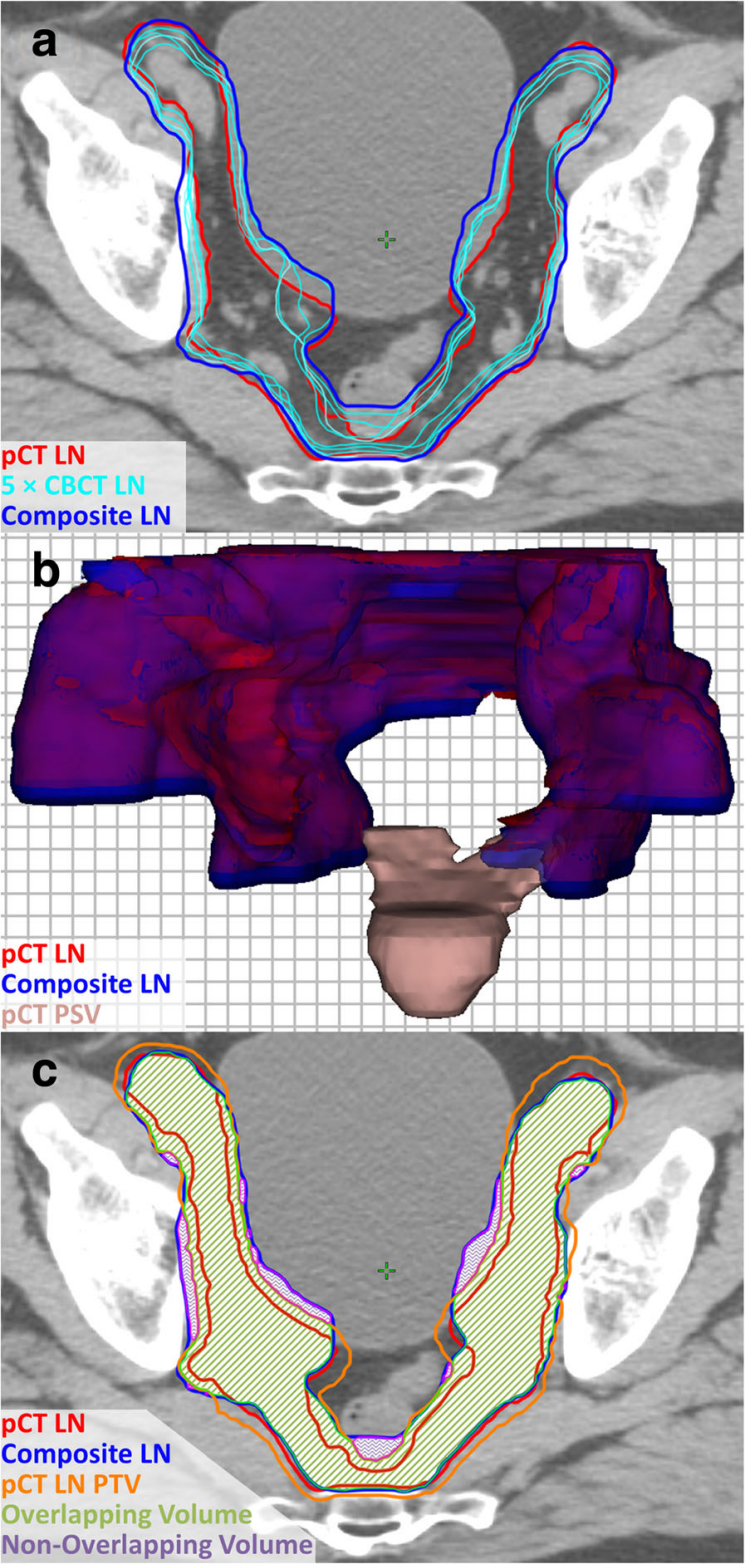
**a** Composite volume generation for soft tissue-registered LN CTV contours. **b** 3D rendering of the original pCT LN CTV and the soft tissue composite LN structure. **c** Overlap analysis for a uniform 3 mm margin

Volumes contoured on each CBCT were transferred to a single pCT structure set. Two datasets per patient were created, which accounted for translational shifts determined from each registration method (i.e., bone and ST matches). Fig. 1a shows an example of LN axial contours from five different CBCTs (cyan) overlaid on the corresponding pCT axial slice; in this example, a ST match to the prostate was used to register each of the CBCT images to the pCT.

The image-registration and contouring tools available within Eclipse™ were used to combine the CBCT contours and to compare the resulting composite structure (blue) to the CTV structures originally delineated on each patient’s pCT (red). CBCT contours that were transferred using the same registration method (bone or ST) were combined to create two separate composite structures. A 3D rendering comparing the original pCT LN CTV structure (red) to the ST-matched composite structure (blue) is also illustrated in Fig. 1b, with the pCT PSV structure (pink) included for reference. The volume of each composite structure was recorded and used for subsequent analysis.

A uniform margin was incrementally increased around the pCT CTV structure (0 to 12 mm; 1 mm increments) to generate a series of PTVs for these structures. For each margin increment, the volume of the overlapping region between the generated PTV and each of the composite structures (bone or ST match) was determined. Fig. 1c shows an example of this analysis on the same axial slice shown in Fig. 1a. In this example, a 3 mm margin has been uniformly extended around the CTV to generate the PTV. Overlapping regions between the PTV and the composite volume are indicated by the green-shaded areas, while the purple-shaded areas indicate non-overlapping regions. The overlapping volume was expressed as a percentage of the composite structure for each PTV margin increment. Additional illustrative examples of this technique are provided in Additional file 1.

### Margin calculation

To correlate with other margin derivation methods [18], a percentage overlap of 95% was selected as the desired threshold criterion. The margins required to achieve 95% overlap for each individual patient and registration option were determined through linear interpolation of the relevant increments.

A population margin was then determined for the 20 patients sampled. Again, as with other techniques, the population margin was defined as the margin required to achieve the desired 95% overlap in 90% of the patient population. For a normal distribution, this can simply be determined from the mean ($\overline{\mathrm{x}}$) and standard deviation (s) of the sampled group, using the formula:

1 $$ \mathrm{Margin}=\overline{\mathrm{x}}+1.28\mathrm{s} $$

PO and PSV margins were then calculated using a commonly used conventional statistical method [18] and compared to those calculated using our composite volume technique. Systematic (Σ) and random (σ) errors, obtained from analysis of a recent audit of our institutional set-up protocol, were employed to determine margins for the two set-up protocols, using the following margin recipe [18]:

2 $$ \mathrm{Margin}=2.5\Sigma +0.7\upsigma $$

It is worth noting that this conventional recipe assumes that treatments consist of a very large number of treatment sessions, each delivering a very small dose fraction, effectively assuming an infinite number of treatment sessions to simplify the mathematical method [18, 21]. These assumptions are clearly not met with SABR treatments and alternative methods have been proposed to address this limitation [21, 22]. However, margin calculations performed using an adapted version of this conventional recipe (VH1 described in [22]) with our derived systematic and random errors agreed well (≤0.3 mm deviation) with the conventional recipe and therefore only the conventional margins are compared with our composite volume method.

Additional data are supplied in the Additional file 2.

### Statistical methods

MATLAB v8.2.0 (MathWorks, Natick, MA) was used to perform a two-sided Wilcoxon signed-rank test to assess the significance in differences between the percentage overlap distributions obtained for each margin increment for the two image-registration matching scenarios (where *p* < 0.05 was considered statistically significant).

For both image-matching protocols, a Shapiro-Wilk normality test was performed using SPSS v22.0.0 (IBM, Armonk, NY), to determine whether the individual margins required to achieve 95% overlap for the 20 patients were normally distributed.

## Results

A total of 120 CT images were individually contoured (20 pCTs and 100 CBCTs). For each target structure (PO, PSV and LN), a minimum of 9 different CTV-PTV margin sizes were analyzed to determine the percentage overlap of the PTV structure with the CBCT composite structures produced for either a bone or ST match.

**Fig. 2:**
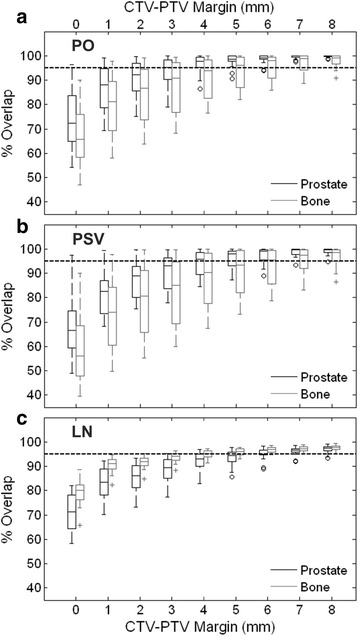
Box-whisker plots of the percentage overlap distributions for the (**a**) prostate (PO) structure, (**b**) prostate and seminal vesicles (PSV) structure, and (**c**) pelvic lymph node (LN) CTV. Differences between the two image-matching protocols were significant for all target structures and margin sizes (*p* < 0.05). The whiskers indicate the last percentage overlap value within 1.5× the interquartile range of its nearest quartile. Individual data points (+/○) represent patient outliers with percentage overlap values outside of this range

The results of the percentage overlap analysis for the three target structures for all 20 patients analyzed are displayed as box-whisker plots in Fig. 2. Differences between the median values of the population overlap distributions for the two registration options (bone or ST) were statistically significant (*p* < 0.05) for all structures and margin sizes investigated.

Table 1 reports the mean and standard deviation of the 95% overlap margin for each of the structures and registration options, as well as additional results obtained from the composite volume analysis. The ratio of the composite to pCT volume was 1.40 and 1.33, 1.57 and 1.44 and 1.22 and 1.33 respectively for bone and ST matches for the PO, PSV and LN structures. As an example, employing a CTV-PTV margin of 5 mm, and using a prostate-based ST match, the average percentage overlap of the composite CBCT volume with the pCT volume was 98.0%, 96.6% and 93.6% for the PO, PSV and LN structures respectively.

**Table 1:** Volume and individual margin characteristics for the pCT target structures and the composite structures generated from contours on the CBCT images

	Structure	PO		PSV		LN	
	Match type	Bone	ST	Bone	ST	Bone	ST
pCT CTV volume (cm^3^)	Average	25.9		36.1		360.2	
	Std. Dev.	10.5		14.1		55.2	
$\frac{\mathrm{Composite}\ \mathrm{volume}}{pCT\kern0.5em \mathrm{volume}}$	Average	1.40	1.33	1.57	1.44	1.22	1.33
	Std. Dev.	0.23	0.24	0.36	0.26	0.16	0.22
% overlap with 5 mm margin	Average	93.4	98.0	90.5	96.6	95.9	93.6
	Std. Dev.	6.64	2.51	8.96	3.72	1.34	3.15
95% overlap margin (mm)	Average ($\overline{\mathrm{x}}$)	4.71	2.83	5.71	3.79	3.93	5.55
	Std. Dev. (s)	2.83	1.87	3.29	2.13	1.50	1.59

Table 2 reports the population CTV-PTV margins for the composite volume and conventional statistical techniques. The composite volume analysis indicated PO population margins of 8.3 and 5.2 mm (s = 2.83 and 1.87 mm) for bone and ST matches respectively. For the PSV structures, calculated population margins were 9.9 and 6.5 mm for the bone and ST matches.

**Table 2:** Population margins calculated using a conventional margin recipe [18] and the composite volume technique for each image-matching scenario

Match type	Technique	Structure	Margin (mm)
Bone	Margin Recipe	PO/PSV	9.4
	Composite volume	PO	8.3
		PSV	9.9
		LN	5.9
Soft Tissue (prostate)	Margin Recipe	PO/PSV	6.7
	Composite volume	PO	5.2
		PSV	6.5
		LN	7.6

The bone-matching protocol required smaller margins around the LN structures, indicating that a margin of 5.9 mm was required to achieve 95% overlap in 90% of the patients. A margin of 7.6 mm was calculated for the LN CTV structure when a prostate-based ST match was performed. Margins calculated using the composite volume method for the PO and PSV structures showed good agreement with the results of the conventional margin recipe. The margin calculated for the PSV structure using a bone-matching protocol was within 0.5 mm of that derived using the statistical method (9.9 and 9.4 mm for the composite volume and conventional techniques respectively).

Additional data for individual patients and margin increments are supplied in the Additional file 3.

## Discussion

A new alternative method to determine planning margins in scenarios where complex multiple-target volumes are treated with a single isocentre has been described. The technique employs a similar methodology to that used by Mak et al. to evaluate seminal vesicle inter-fraction motion and its relationship to rectal and bladder filling [23]. The current methodology allows derivation of set-up margins but could potentially be adapted to include delineation and intrafraction motion errors through the use of additional contours drawn by independent observers and including contours from post-treatment CBCT images [24]. Margins included soft tissue geometrical information obtained from repeat CBCT images as opposed to translational registration errors used traditional margin recipes.

Using a prostate-based ST match, this composite volume analysis yielded margins of 5.2, 6.5 and 7.6 mm for PO, PSV and LN volumes respectively. The PO and PSV margins showed good agreement with PO/PSV margins derived using a conventional statistical margin recipe that included delineation uncertainty and intrafraction motion errors [18]. These margins were also very similar to the lower limit values determined by Oehler et al., who reported CTV-PTV margins of between 5 and 8 mm for PO and 6-11 mm for PSV structures, again using the conventional method with delineation uncertainty and intrafraction motion errors included [24].

As expected, this new analysis indicated that matching to the prostate requires relatively smaller PO and PSV margins and a larger margin around the LN CTV, thus facilitating margin reduction around the high-dose targets and minimising the volume of normal tissue receiving higher doses of radiation. Many institutions commonly include the PSV and LN when treating men with high-risk localized PC [25, 26]. It is therefore important to accurately quantify the CTV-PTV margins required for each target structure when matching to the prostate structure as small changes have the potential to cause much larger shifts in the LN CTV [15, 27].

A number of studies have reviewed LN margins in the context of conventionally fractionated prostate and pelvic radiotherapy, each adopting a different approach and consequently reporting a range of nodal CTV-PTV margins. For example, Ferjani et al. mapped original planning structures to selected CBCTs using bone and prostate matches for six patients [16]. They then applied the original IMRT plan (recalculated without heterogeneity correction) to estimate the dose based on both matches for a given CBCT. They concluded that CTV-PTV margins of 8 mm (6 mm posteriorly) to the prostate and 5 mm to the LN were sufficient for concurrent treatment with CBCT prostate-based matching.

By evaluating relatively more image sets for fewer patients than our study, Ferjani et al.’s results provide a better indication of intra-patient variability. However, by including a larger sample size (*n* = 20) and manually delineated individualized CTVs for each CBCT, our study provides a strong indication of inter-patient variability and a more accurate representation of the true treatment anatomy. Additionally, given the uncertainties inherent in CBCT-based dose modeling [28], we elected to pursue a purely anatomy-based approach.

The 5 mm LN margin recommended by Ferjani et al. is substantially smaller than the ≥13 mm “vascular space” margin recommended by Wang et al., which was also based on a prostate-matching regime [14]. Wang et al.’s margin was derived by mapping three separate IMRT plans (with varying CTV-PTV margin) onto serial CBCTs for eight patients. The dose computed on each CBCT was subsequently mapped back to the original pCT and summed to generate DVHs for each structure of interest which were analyzed to determine the optimum margin.

Hinton et al. employed another technique which used measured couch shifts to derive nodal CTV-PTV margins of 9 mm in the anterior-posterior direction and 7 mm laterally [15]. These margins are similar (<1.4 mm difference) to those calculated in our study which considered an isotropic margin expansion in all three Cartesian planes as analysis of each patient’s composite volume indicated comparable structure motion in all three directions.

With regard to SABR, in a single study, Kishan et al. used fiducial-based CBCT matching to evaluate 12 patients [17]. Selected CBCTs were registered to the pCT, allowing transfer of dose distributions and the original pCT contours. They found that standard LN margins of 4-5 mm were acceptable, under the conditions that the superior displacement of the prostate was kept to ≤5 mm and the relative change in bladder height was <18%. This margin is considerably smaller than the 7.6 mm calculated from our analysis, which avoided CBCT dose calculation uncertainty and used delineated structures based on actual CBCT anatomy.

The authors acknowledge that there are some limitations to the current study. Firstly, post-treatment CBCT, pitch / roll and rotational corrections and real-time tracking of the prostate were not incorporated, reflecting current clinical practice in our and many other institutions. However, available data indicate that these are largely accounted for by conventional margin expansion in SABR [29–31] and future extensions to the technique are planned to confirm this. Secondly, the ST resolution with CBCT is poorer than with conventional CT, particularly with regard to delineation of the prostate-rectum interface [32–34]. This issue, in conjunction with potential errors with image-matching, is an inherent feature of this type of study [35, 36]. In this investigation, CBCTs of insufficient image quality were not included in the analysis and a single experienced uro-oncologist (CL) contoured and matched all CTs following a well-defined protocol [19], and a second clinical oncologist (SJ) peer-reviewed the resulting structures and registrations. While contouring of serial CBCTs is currently a time-intensive process that is not routinely implementable into clinical workflow, rapid advances in auto-contouring algorithms may facilitate wider adoption of this method. Finally, due to the inherent difficulties in performing accurate dose calculations using CBCT [37], this study only evaluated geographic changes and did not include a dosimetric analysis.

Particular strengths of this study include the sample size and number of CBCTs evaluated, the use of individually contoured LN CTVs for pCTs and CBCTs, and the independence of the composite volume CTV-PTV margin derivation method from the assumptions of conventional margin recipes. In many previous studies, couch shifts [38], representative slices [15] or the original nodal CTV alone [39] were chosen to represent serial nodal CBCT CTVs. In addition, the superior-inferior CTV-PTV margin was not accounted for in some cases [15]. In this study, the entirety of each volume was considered in all three planes. Individual protocol-based CTVs were generated for all 100 CBCTs in a more accurate reflection of the true anatomical situation during treatment. This is particularly important in the case of the LN CTV, due to the greater impact of variability of the size and position of the OARs. The results of this analysis will be utilized in an institutional trial of prostate and pelvic nodal SABR in men with high-risk localized PC [13]. However, this method could also be applied to other sites where multiple target volumes are treated with a single isocentre.

## Conclusions

Current methods of CTV-PTV margin calculation for conventional radiotherapy may not be sufficient for the derivation of margins in the setting of SABR and/or multiple isocentric CTVs. We have presented a novel method of CTV-PTV margin derivation that is applicable to the single isocentre treatment of more than one target volume and/or SABR. When applied to prostate and seminal vesicle target structures, which are a good facsimile for the target geometries assumed in conventional margin recipes, this method yielded comparable results to conventional methods. Margins calculated from this analysis have been used to inform an institutional prostate and pelvic nodal SABR trial.

## Additional files

Additional file 1: Appendix A: PTV Expansion Percentage Overlap Example. (DOCX 1032 kb)

Additional file 2: Appendix B: Derivation of Population Margins. (DOCX 75 kb)

Additional file 3: Appendix C: Margin expansion results for individual patients. (XLSX 35 kb)

## Abbreviations

CBCT: Cone-beam CT
CT: Computed tomography
CTV: Clinical target volume
DVH: Dose-volume histogram
kV: Kilovoltage
LN: Lymph nodes
OAR: Organs at risk
PC: Prostate cancer
pCT: Planning CT
PO: Prostate
PSV: Prostate and seminal vesicles
PTV: Planning target volume
SABR: Stereotactic ablative radiotherapy
ST: Soft tissue
