# Supplementary material for: A novel CBCT-based method for derivation of CTV-PTV margins for prostate and pelvic lymph nodes treated with stereotactic ablative radiotherapy
Source: Radiat Oncol. 2017 Aug 4;12:124. doi: 10.1186/s13014-017-0859-z (PMC5543558; doi:10.1186/s13014-017-0859-z)
Supplement: Supplementary file 1 — Appendix A: PTV Expansion Percentage Overlap Example. (DOCX 1032 kb) [file 13014_2017_859_MOESM1_ESM.docx]

# Appendix A: PTV Expansion Percentage Overlap Example

Figure A.1 displays additional examples of the effect of increasing the CTV-PTV margin on the composite volume analysis. These examples use the same patient data presented in Figure 1. The same colour properties as Figure 1 have also been used to indicate structures of interest, with the overlap regions between the soft tissue composite volume and the PTV structure indicated by the shaded green area.

As expected, larger percentage overlap between the two structures is observed with increasing margin size, with 97.0% overlap calculated for a 4mm margin and 98.8% overlap recorded for an 8mm margin (Figure A.1 (c)). However, it is clear from Figure A.1 (c) that a large amount of normal tissue is also encompassed within this 8 mm margin.


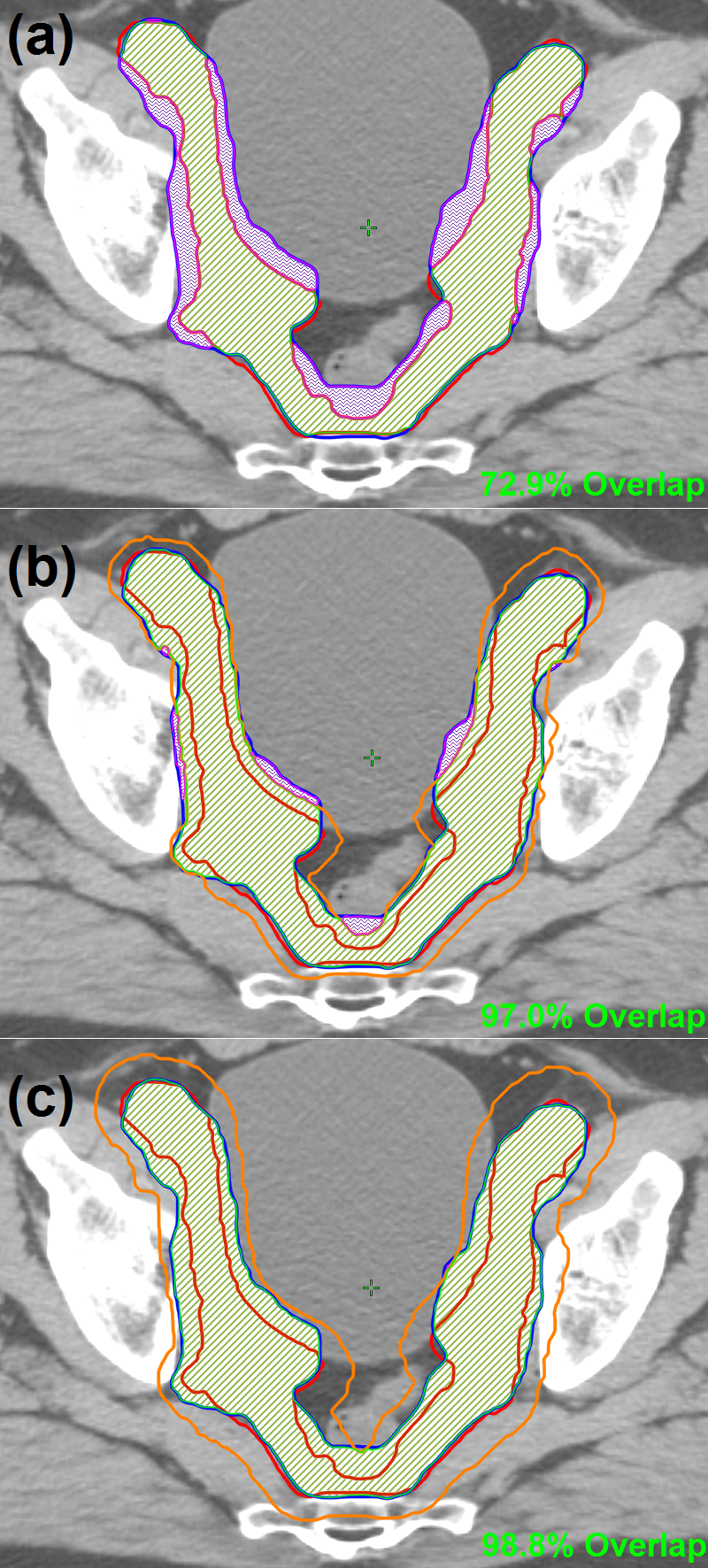


Figure A.1 Overlap analysis for increasing CTV-PTV margin increments. (a) 0 mm margin. (b) 4 mm margin. (c) 8 mm margin.
