# Supplementary material for: A novel CBCT-based method for derivation of CTV-PTV margins for prostate and pelvic lymph nodes treated with stereotactic ablative radiotherapy
Source: Radiat Oncol. 2017 Aug 4;12:124. doi: 10.1186/s13014-017-0859-z (PMC5543558; doi:10.1186/s13014-017-0859-z)
Supplement: Supplementary file 2 — Appendix B: Derivation of Population Margins. (DOCX 75 kb) [file 13014_2017_859_MOESM2_ESM.docx]

# Appendix B: Derivation of Population Margins

# Composite Volume Analysis

Normality tests performed on the distributions of the 95% overlap margin did not reject the null hypothesis that they were normally distributed. Equation 1 in the main text was therefore employed to calculate the margin required to achieve 95% overlap in 90% of the patient population, using the mean and standard deviation results reported in Table I of the main text.

For a normally distributed population, the percentage of the population that should achieve 95% overlap with a composite structure for a specific margin size can be estimated from the mean (x̄) and standard deviation (s) of the distribution. As indicated in Figure B.1, 50% of the population will achieve 95% overlap if x̄ is used as the CTV-PTV margin. A margin of x̄ + s will result in approximately 84% achieving 95% overlap, while a margin of x̄ + 1.28s is required for 90% of the population to achieve 95% with the composite structures.


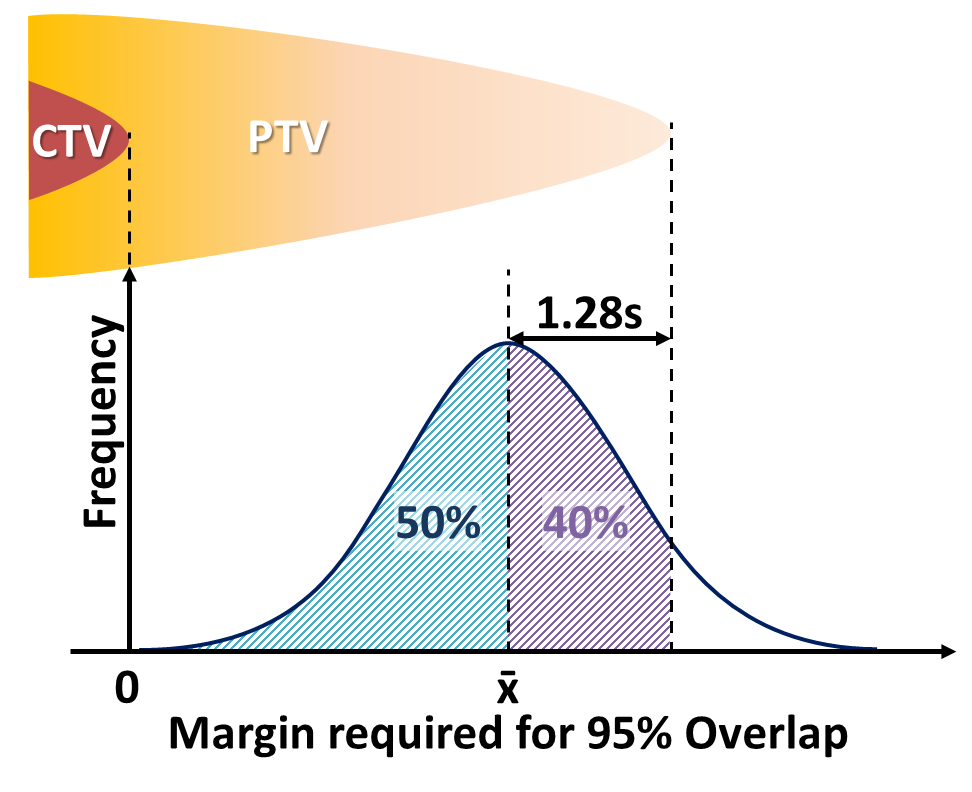


Figure B.1 Sketch illustrating derivation of margin required for 90% of the population to achieve 95% overlap in a normally distributed sample.

## Prostate CTV-PTV Margin Calculation: Statistical Method

As part of a local audit of our centre’s set-up protocol, CBCT images from 20 prostate IMRT patients were analysed following their treatment. Our current imaging protocol states that, following the patient’s set-up, CBCT images should be acquired for the first 3 fractions and on a weekly basis subsequent to this. Registration of the CBCT images to the patient’s pCT is performed, accounting for translational shifts only, using the patient’s bony anatomy as a surrogate for the CTV.

Post-treatment CBCT’s were also acquired for this sample of patients for a number of their treatments (range 5 – 10), these were registered with the pCT and saved for analysis offline. Following treatment, the saved CBCT images (249 pre-treatment and 130 post-treatment) were reviewed offline by a single clinical oncologist (CL) using the Aria Offline Review program (Varian Medical Systems). For the pre-treatment images, additional matches were carried out for the PSV CTV volume. Offline matching was also performed on the post-treatment images for the same structures of interest (i.e. bone surrogate and PSV).

The results of this matching study were analysed to derive systematic (∑) and random (σ) uncertainties for various sources of uncertainty. The results of this analysis were employed in the conventional van Herk CTV-PTV margin formula [14]:

|  | $\mathbf{Margin}\boldsymbol{=2.5}\boldsymbol{\Sigma}\boldsymbol{+0.7}\boldsymbol{\sigma}$ | 1 |
| --- | --- | --- |

For comparison to the composite volume analysis presented in this article, margins based on two online registration protocols were calculated using variants of this formula.

The formula used for the bone matching protocol was given by:

|  | $\mathbf{Margin}=\mathbf{2}.\mathbf{5}\left( \sqrt{\boldsymbol{\Sigma}_{\boldsymbol{Delin}}^{\mathbf{2}}+\boldsymbol{\Sigma}_{\boldsymbol{Inter}}^{\mathbf{2}}+\boldsymbol{\Sigma}_{\boldsymbol{Intra}}^{\mathbf{2}}} \right)+\mathbf{0}.\mathbf{7}\left( \sqrt{\boldsymbol{\sigma}_{\boldsymbol{Inter}}^{\mathbf{2}}+\boldsymbol{\sigma}_{\boldsymbol{Intra}}^{\mathbf{2}}} \right)$ | 2 |
| --- | --- | --- |

Where subscripts indicate the source of error: Delin = delineation error, obtained from external studies [24]; Inter = Interfraction motion of the PSV structure relative to the bony surrogate and Intra = intrafraction motion of PSV, determined from fractions with pre- and post-treatment CBCTs.

A reduced equation was used to determine margins for the soft tissue registration protocol, where residual interfraction error was not considered:

|  | $\mathbf{Margin}\boldsymbol{=2.5}\left( \sqrt{\boldsymbol{\Sigma}_{\boldsymbol{Delin}}^{\boldsymbol{2}}\boldsymbol{+}\boldsymbol{\Sigma}_{\boldsymbol{Intra}}^{\boldsymbol{2}}} \right)\boldsymbol{+0.7}\boldsymbol{\sigma}_{\boldsymbol{Intra}}$ | 3 |
| --- | --- | --- |

Table B.1 reports the systematic and random errors calculated from the analysis of the CBCT images obtained for the patient group used in our clinical audit, including values for systematic delineation errors obtained from literature [18].

Table B.1 Systematic and random geometric errors obtained from literature or calculated from analysis of CBCTs acquired during the pilot study.

| Error Source | Error Type | Uncertainty (mm) | | |
| --- | --- | --- | --- | --- |
|  |  | Ant-Post | Sup-Inf | Lateral |
| Delineation [18] | Σ_Delin_ | 1.52 | 2.03 | 1.68 |
| Interfraction Motion | Σ_Inter_ | 1.897 | 2.103 | 0.646 |
|  | σ_Inter_ | 1.975 | 1.758 | 0.884 |
| Intrafraction Motion | Σ_Intra_ | 0.922 | 0.942 | 1.075 |
|  | σ_Intra_ | 1.542 | 1.610 | 1.347 |

Tables B.2 and B.3 report the margins calculated using these uncertainty values for the online bone and soft-tissue matching protocols respectively.

Table B.2 Margin calculation for online bone matching protocol

| Axis | Error Type | (Uncertainty)^2^ | | | Sum | $\sqrt{\mathrm{Sum}}$ | Margin |
| --- | --- | --- | --- | --- | --- | --- | --- |
|  |  | Delineation | Inter | Intra |  |  |  |
| Ant-Post | Systematic | 2.31 | 3.60 | 0.85 | 6.76 | 2.60 | 8.3 |
|  | Random |  | 3.90 | 2.38 | 6.28 | 2.51 |  |
| Sup – Inf | Systematic | 4.12 | 4.42 | 0.89 | 9.43 | 3.07 | **9.3** |
|  | Random |  | 3.09 | 2.59 | 5.68 | 2.38 |  |
| Lateral | Systematic | 2.82 | 0.42 | 1.16 | 4.40 | 2.10 | 6.4 |
|  | Random |  | 0.78 | 1.81 | 2.60 | 1.61 |  |

Table B.3 Margin calculation for online soft-tissue matching to PSV protocol

| Axis | Error Type | (Uncertainty)^2^ | | Sum | $\sqrt{\mathrm{Sum}}$ | Margin |
| --- | --- | --- | --- | --- | --- | --- |
|  |  | Delineation | Intra |  |  |  |
| Ant-Post | Systematic | 2.31 | 0.85 | 3.16 | 1.78 | 5.5 |
|  | Random |  | 2.38 | 2.38 | 1.54 |  |
| Sup – Inf | Systematic | 4.12 | 0.89 | 5.01 | 2.24 | **6.7** |
|  | Random |  | 2.59 | 2.59 | 1.61 |  |
| Lateral | Systematic | 2.82 | 1.16 | 3.98 | 1.99 | 5.9 |
|  | Random |  | 1.81 | 1.81 | 1.35 |  |
